# Aptitude and attitude: predictors of performance during and after basic laparoscopic skills training

**DOI:** 10.1007/s00464-021-08668-8

**Published:** 2021-08-09

**Authors:** Kirsty L. Beattie, Andrew Hill, Mark S. Horswill, Philip M. Grove, Andrew R. L. Stevenson

**Affiliations:** 1 School of Psychology, The University of Queensland, Brisbane, Australia; 2 Clinical Skills Development Service, Metro North Hospital and Health Service, Brisbane, Australia; 3 Minerals Industry Safety and Health Centre, Sustainable Minerals Institute, The University of Queensland, Brisbane, Australia; 4 School of Medicine, The University of Queensland, Brisbane, Australia; 5 Department of Colon and Rectal Surgery, Royal Brisbane and Women’s Hospital, Brisbane, Australia

**Keywords:** Laparoscopy, Skill, Ability, Potential, Training, Psychology

## Abstract

**Background:**

Manual dexterity and visual-spatial ability are considered key to the development of superior laparoscopic skills. Nevertheless, these abilities do not reliably explain all the variance found in the technical performance of surgical trainees. Consequently, we must look beyond these abilities to improve our understanding of laparoscopic skills and to better identify/develop surgical potential earlier on.

**Purpose:**

To assess the individual and collective impact of physical, cognitive, visual, and psychological variables on performance during and after basic simulation-based laparoscopic skills training.

**Method:**

Thirty-four medical students (laparoscopic novices) completed a proficiency-based laparoscopic skills training program (using either a 2D or 3D viewing mode). This was followed by one testing session, a follow-up testing session with new (yet similar) tasks, and a series of physical, cognitive, visual, and psychological measures.

**Results:**

The statistical models that best predicted variance in *training* performance metrics included four variables: *viewing* *mode* (*2D* vs *3D*), *psychological* *flexibility*, *perceived* *task* *demands*, and *manual* *dexterity* (*bimanual*). In subsequent *testing*, a model that included *viewing* *mode* and *manual* *dexterity* (*assembly*) best predicted performance on the pre-practiced tasks. However, for a highly novel, spatially complex laparoscopic task, performance was best predicted by a model that comprised *viewing* *mode*, *visual-spatial* *ability*, and *perceived* *task* *demands*. At follow-up, *manual* *dexterity* (*assembly*) alone was the best predictor of performance on new (yet similar) tasks.

**Conclusion:**

By focussing exclusively on physical/cognitive abilities, we may overlook other important predictors of surgical performance (e.g. psychological variables). The present findings suggest that laparoscopic performance may be more accurately explained through the combined effects of physical, cognitive, visual, and psychological variables. Further, the results suggest that the predictors may change with both task demands and the development of the trainee. This study highlights the key role of psychological skills in overcoming initial training challenges, with far-reaching implications for practice.

## Background

With an exponential growth in minimally invasive procedures and a shift towards shorter workweeks in many countries, surgical residents now face a greater complexity of practice with potentially fewer opportunities for operational experience [1, 2]. While robotic approaches may help to overcome the various learning difficulties associated with minimally invasive surgery, the development of laparoscopic skills remains fundamental to achieving safe and efficient outcomes. Nevertheless, trainees have been found to acquire laparoscopic skills at different rates, with 8–20% failing to reach proficiency in simulation-based laparoscopic skills training, regardless of continued practice [3–5]. As a result, it is essential that the chosen few possess the necessary knowledge, skills, abilities, and attributes to reach laparoscopic proficiency with greater efficiency and efficacy. However, the most important predictors of surgical potential are still a point of conjecture and debate. While research shows that academic achievements (e.g. grades, test knowledge) are limited in their ability to predict surgical performance [6–8], many surgeons believe that certain innate abilities are the key to superior laparoscopic skill [7].

## Innate abilities

When 58 master surgeons were asked to identify the most important attributes for surgical trainees, *innate* *dexterity* was reported as the strongest perceived predictor of technical skill in both training and practice [7]. This incorporated psychomotor abilities such as hand–eye coordination, limb coordination, speed, and steadiness (i.e. *manual* *dexterity*), as well as spatial perception, and the ability to visually interpret and manipulate images (i.e. *visual-spatial* *ability*) [7]. Moreover, these abilities were considered particularly vital for the development of laparoscopic skills to manage the visuomotor discordance and increased technical complexity created by the fulcrum effect (i.e. the counterintuitive movements required by the perceived inversion of motion from the handle to the end of the instrument) [7, 9].

Empirical research has since shown significant correlations of both manual dexterity and visual-spatial ability with novices’ technical performance (i.e. efficiency and accuracy) in laparoscopic skills training [10–15]. While these relationships have not always been observed [16, 17], inconsistent and conflicting results may be due to various methodological limitations common in this research field. For example, many studies have neglected to assess or control for potentially confounding visual variables (e.g. visual acuity, stereoacuity) that may impact laparoscopic performance, particularly with different viewing modes (i.e. 2D and 3D) [18, 19]. Furthermore, past research has often focussed on the independent effects of manual dexterity [17], visual-spatial ability [20], and visual ability [21], yet their combined effects have been found to explain a greater portion of variance in laparoscopic performance [22, 23]. While design differences may account for some of the inconsistent findings throughout the literature, variance still exists in the performance of surgical trainees that cannot be explained by the individual or combined effects of such innate abilities [22]. Consequently, it is important to expand beyond the scope of past research and consider other individual differences that may account for further variance in performance and enhance the prediction of surgical potential.

## Perceived demands

Research has shown that higher levels of stress (i.e. where internal/external demands are perceived by the individual as a *threat* rather than a *challenge*) are associated with poorer surgical performance (i.e. longer times and increased errors), and inferior economy of motion during laparoscopic skills training and practice [24–26]. Excessive levels of stress have also been found to disrupt and relocate limited attentional resources and impair visual-spatial ability, hand-eye coordination, memory, situation awareness, and decision-making in various high-stakes contexts [27–29]. Such results suggest that a surgeon’s innate physical and cognitive abilities may be significantly impacted by their psychological state and the way they perceive internal and external demands [25]. For example, if a surgeon becomes overwhelmed at critical points of complexity and pressure, their *normally* quick hand movements and cognitive processing may be interrupted or impaired.

To address these concerns, many researchers have attempted to predict how candidates will work under pressure by defining the ideal psychological states, traits, characteristics, behaviours, and/or coping strategies of practicing surgeons [30–38]. However, the results have ultimately lacked clarity, consistency, and an objective and reliable connection with technical skill [39, 40]. It is suggested that such limitations are likely due to the ill-defined labels used to identify levels of expertise (e.g. the skills and abilities of a “master surgeon” have yet to be defined), and the inaccurate assumptions that years of surgical experience reflect a particular level of surgical skill [41]. More importantly, this area of research has largely overlooked *why* individuals subjectively evaluate the same demand in different ways (e.g. why one individual sees it as a challenge, while another sees it as a threat), and *how* this impacts subsequent performance [42]. As a result, we must look beyond the surgical literature to better understand the key psychological mechanisms behind perception and performance in this high-stress and high-demand contexts.

## Psychological flexibility

In other high-stakes fields such as aviation, the military, and elite sports, an attribute known as *psychological* *flexibility* has been consistently found to impact well-being, stress, technical and non-technical performances, and skill development under pressure [43–48]. For example, greater psychological flexibility has been associated with increased resilience and reduced risk of posttraumatic stress in active Air Force personnel, improved decision-making in aircrew, and higher coach-ratings of athlete’ performances [43–48]. Psychological flexibility can be described as the ability to remain aware and engaged in one’s present experience without internal distraction, while persisting or changing actions towards chosen goals or values [46, 49]. It is important to note that psychological flexibility is not simply a state of happiness, well-being, positivity, or ease, but rather involves *functional* responses to difficult, uncertain, and challenging thoughts/feelings/situations [50]. Furthermore, improving psychological flexibility is the core function of mindfulness-based practice (a growing area involving stress-reduction techniques) [51–53], which is considered to benefit performance by reducing the attentional resources spent trying to change, control, judge, or avoid internal thoughts or events [54]. This then leaves greater awareness available for task-relevant cues and more effective responding to contingencies in the environment [54]. As surgical excellence is not marked by error-free performance, but rather the ability to manage errors, disruptions, and complications, and efficiently change techniques and strategies to meet these challenges [55, 56], it is suggested that a surgeon’s psychological flexibility may carry significant implications in the operating theatre.

While it appears that psychological flexibility has yet to be explored in a surgical context, it has been associated with increased emotion regulation, self-efficacy, resilience, and work performance in other health professions [57–59]. Additionally, Lebares et al. (2019) [60] found that surgical residents who completed laparoscopic skills training in combination with a mindfulness-based training intervention, showed improved well-being, executive functioning, and motor performance at a 1-year follow-up, compared to residents who completed laparoscopic skills training alone. This suggests that more mindful and flexible behaviours may enhance surgical performance by optimising attentional resources and reducing the deleterious effects of stress. This may also help to explain *why* certain individuals perceive and react to demands in different ways, and *how* this changes their performance. However, this point remains conjecture given the lack of investigation into psychological flexibility within a surgical context. Also, to the authors’ knowledge, no published empirical study has explored psychological variables in combination with physical, cognitive, and visual abilities to examine their individual and collective impact on laparoscopic skills.

## Aims

To expand beyond past research, the study had two main aims. The first aim was to assess the individual and collective impact of (a) manual dexterity, (b) visual-spatial ability, and (c) visual abilities (i.e. visual acuity and stereoacuity) on performance in proficiency-based laparoscopic skills training and testing (while controlling for viewing mode: 2D/3D). The second aim was to assess the individual and collective impact of (d) perceived demands and (e) psychological flexibility on performance in proficiency-based laparoscopic skills training and testing (beyond that of other variables). By enhancing our ability to predict surgical potential, it may be possible to equip medical students to make more informed decisions about their suitability for a surgical career early on, and allow them to focus on developing all relevant supportive skills/attributes prior to formal selection and training. Moreover, by the time they reach surgical residency, applicants may be more likely to possess the necessary aptitude and attitude to reach laparoscopic proficiency with increased efficiency and efficacy.

## Method

### Participants

Thirty-four laparoscopic novices (male, *n* = 18; female, *n* = 16) with a mean age of 25.29 years (*SD* = 3.66, range 19–34) voluntarily participated in this study. Participants were current medical students (between the first and fourth year of medical school), recruited from a larger sample who participated in Beattie et al.’s (2020) [61] study investigating the effects of 2D vs 3D viewing modes on laparoscopic skills training. Thirty-two participants were right-handed and two were left-handed. Participants received no financial compensation for their involvement. The study was approved by the Royal Brisbane and Women’s Hospital (RBWH) and the University of Queensland ethical committees.

### Materials and measures

#### Laparoscopic tasks

*Training* *tasks* Participants performed all six tasks from the 3-Dmed program (3-Dmed®, Franklin, OH, US) to a baseline level of proficiency (see Table 1) using a box trainer and laparoscopic instruments. Total scores were calculated by the total time taken (in seconds), combined with the total time penalties incurred for errors across all training repetitions (in seconds). Consequently, lower scores reflected more efficient and accurate performances. All baseline levels of proficiency, error parameters, and instructions outlined by Schreuder et al. (2011) [62] were adopted in this research (see Table 1 for an overview of the six tasks).

**Table 1:** Overview of the 3-Dmed tasks used in training and testing (parameters adopted from Schreuder et al. 2011)

Task	Proficiency Score (in seconds)	Penalties	Description
1. Post and sleeve	120	Drop sleeve *on* the board = 10 s Drop sleeve *off* the board = 20 s	Pick up and transfer six sleeves from one side of the board to the other, and back again, using both hands
2. Loops and wire	86	Miss a loop = 10 s	Feed two pipe cleaners through two rows of loops using both hands, starting one from the left side and one from the right side
3. Pea on a peg	313	Drop bead *on* the board = 10 s Drop bead *off* the board = 20 s	Pick up wooden beads from a cup and place them onto 14 pegs, using the right hand to complete the right side and the left hand to complete the left side
4. Wire chaser (one hand)	69	Lose/drop the ring = 10 s	Move three rings of decreasing diameter to the other end of a curved wire with one hand
5. Wire chaser (two hands)	127	Lose/drop the ring = 10 s	Move three rings of decreasing diameter to the other end of a curved wire with two hands
6. Zig-zag loop	48	Miss a loop = 10 s	Alternately feed a rope through the first and second rows of loops using both hands to make an “M”-shaped or zigzag pattern

*Testing* *tasks* During testing, participants completed all 3-Dmed tasks again (twice) with the same scoring parameters, followed by a novel task developed and validated by Sakata et al. (2017) [18, 63], known as the “Navigating in Space” task (NIS). The NIS task assesses fine dexterity and required participants to pass a curved suture through six flexible loops (made from a monofilament strand) in a pre-defined sequence with two needle holders. Scores on the NIS task were calculated by the total time taken (in seconds) to complete the last two repetitions of the task (with a time limit of 10 min per attempt). The first attempt was used to familiarise participants with the task requirements.

*Follow-up* *tasks* In the follow-up session, participants completed the first two tasks from the FLS training program (i.e. Peg Transfer and Precision Cutting) three times each. Similar to the first 3-Dmed task (i.e. Post and Sleeve), the Peg Transfer task required participants to pick up and transfer six objects from one side of the board to the other, and back again, using both hands. The Precision Cutting task required participants to use a grasper in one hand to provide traction to a piece of gauze, and endoscopic scissors in the other hand to cut around a pre-marked double-circle on the gauze. All standard instructions and scoring parameters defined by the FLS program (https://www.flsprogram.org/) were adopted here.

#### Innate abilities

With manual dexterity, visual-spatial ability, visual acuity, and stereoacuity found to significantly correlate with laparoscopic training performance [13–15, 18], participants were assessed on these variables as baseline measures of physical, cognitive, and visual ability in the screening session. The Purdue Pegboard (PP) test (Lafayette Instrument Co) was used to measure innate manual dexterity through four sub-tests: right hand (alone), left hand (alone), bimanual (both hands together), and an assembly task (both hands working simultaneously and continuously to construct assemblies with multiple elements). Higher scores on the PP reflected superior manual dexterity. An online version of the Mental Rotations Test was used to assess participants’ visual-spatial ability, with higher scores reflecting more efficient and accurate visual-spatial ability [64]. Participants’ right and left visual acuity, and their level of stereoacuity were also assessed using the LogMAR eye chart (National Vision Research Institute, Melbourne, Australia) and Randot Stereo Test (Stereo Optical, Chicago, IL), respectively. In both these tests, lower scores reflected a greater level of visual ability.

#### Perceived demands

With high levels of stress found to negatively impact laparoscopic performance [65], participants completed the well-validated NASA Task Load Index (NASA-TLX) [66, 67] at the end of each session to assess the level of mental demand, physical demand, temporal demand, performance, effort, and frustration/stress that they associated with the tasks. Participants rated each of these dimensions on a 7-point scale [68] from *Very* *Low* (scored 0%) to *Very* *High* (scored 100%), except for performance, which was anchored at *Perfect* (scored 0%) and *Failure* (scored 100%). Lower scores on the index indicated less demand/stress perceived from the tasks.

#### Psychological flexibility

Participants’ level of psychological flexibility was measured using the validated 7-item Acceptance and Action Questionnaire-II (AAQ-II; [69]). An example statement from the AAQ-II includes “Worries get in the way of my success”. Each statement required a response on a 7-point scale from 1 (*never* *true*) to 7 (*always* *true*). All responses were reversed scored so that higher scores reflected a higher level of psychological flexibility, and lower scores reflected greater experiential avoidance (or inflexibility).

Despite being recruited through a surgical interest group (requiring self-selection), participants’ “interest in surgery compared to the average medical student” was also measured on a 5-point scale from *Very* *Low* (1) to *Very* *High* (5), to confirm if the sample adequately represented those interested in a surgical career. While other exploratory variables were also included as part of a larger body of research (e.g. level of medical study, video-game use; see Beattie et al. 2020 [61]), they were not the focus of the current study (and did not display any significant relationships with performance), thus are not discussed here.

### Procedure

**Fig. 1:**
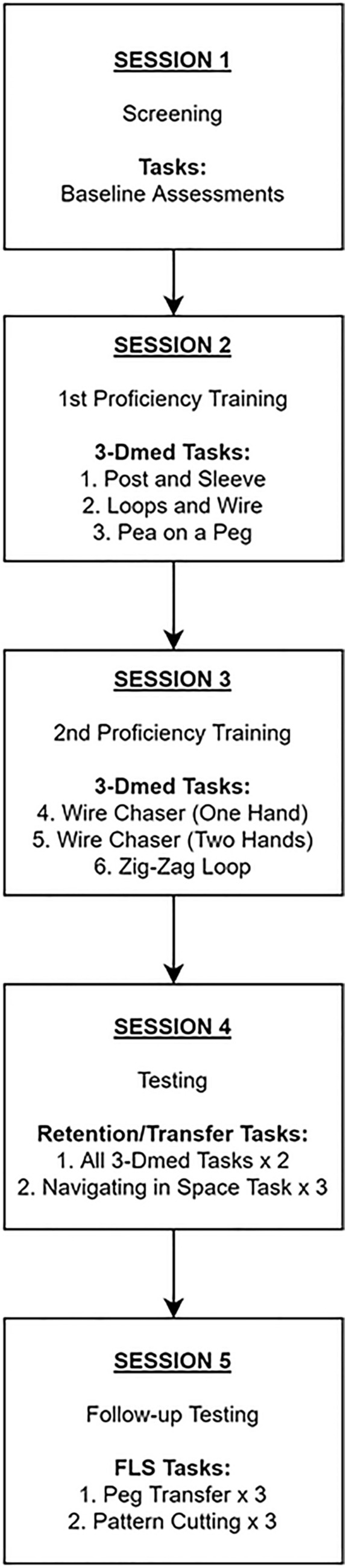
Overview of study design

Participants attended five sessions at a simulation training centre within a tertiary hospital campus, and the first four sessions overlapped with the study conducted by Beattie et al. (2020) [61]. See Fig. 1 for an overview of the complete study design. The first session included the collection of background information and baseline measures of physical, cognitive, and visual abilities. The second and third sessions included the set practice of six laparoscopic skills tasks to a pre-defined level of proficiency using either a 2D or 3D viewing mode. The 3D viewing mode was included to control for its effects on performance (given 3D has the potential for more common use in future due to its efficacy in training) [18, 61]. The fourth session included testing of the six tasks again (twice) and three attempts at a novel task. See Beattie et al. (2020) [61] for further details of the methodology and materials involved.

After completing the fourth session, all participants were invited to participate in a fifth (follow-up) session that was unique to the current study. During this fifth session, participants completed the first two tasks from the Fundamentals of Laparoscopic Skills (FLS) training program (to which they had no prior exposure) three times each in the 2D viewing mode. These tasks were followed by a series of psychological and behavioural measures administered online via the Qualtrics survey platform. It is noted that 23 (out of the 34) participants completed the psychological and behavioural measures via Qualtrics using a laptop in the simulation centre. However, due to COVID-19 restrictions on face-to-face research at the time of the study, 11 participants completed the same psychological and behavioural measures via Qualtrics outside of the simulation centre and could not undertake the two FLS tasks in the follow-up session.

### Statistical analysis

Statistical analyses were conducted using SPSS® version 25 (IBM Corp, 2017, Armonk, NY: USA), with *α* set at 0.05. Independent *t*-tests were run to check for any significant differences between participants who completed the online psychological/behavioural measures in the lab versus outside the lab. To identify the variables for inclusion in multivariate analyses, bivariate correlations among all potential predictor and criterion variables were assessed using Pearson’s correlation coefficients (see Table 2). Any predictor variable that did not have a significant relationship (*p* > 0.05) with any of the criterion variables was excluded from further analyses. After satisfying the mathematical assumptions for multivariate analyses [70], a series of combined hierarchical stepwise regressions was conducted to predict the individual and collective impact of the associated variables on each criterion, respectively (i.e. training repetitions, total training score, total test score, total NIS score, and total follow-up test score). To control for its already well-established effects on laparoscopic performance [61, 71, 72], the viewing mode variable (coded as 2D = 0 and 3D = 1) was entered into Step One of each analysis (except for follow-up testing where participants used 2D only) using the Enter method. Given the exploratory nature of this study, and to avoid overfitting the model, all significantly associated predictor variables were then entered into Step Two using the Stepwise method (to determine the line of best fit with inclusion criteria set at *p* < 0.05, exclusion at *p* > 0.10, and order based on explanatory power) [70]. Despite the relatively small *N* for the number of predictors employed, the sample size per variable still met the minimum requirements suggested in the literature [73, 74]. Additionally, the adjusted *R*^*2*^ is reported here (in addition to the conventional *R*^2^ in Tables 3 - 7) to further account for the relatively small sample size (as suggested by Austin and Steyerberg, 2015 [73]).

**Table 2:** Pearson correlation coefficients between all potential predictor and outcome variables

Variable	M (SD)	1. Training repetitions	2. Total training score	3. Total test score	4. NIS score	5. Total follow-up score^a^	6. 2D/3D viewing mode (training)	7. 2D/3D viewing mode (testing)	8. Manual dexterity (bimanual)	9. Manual dexterity (assembly)	10. Visual spatial ability	11. Visual acuity (L + R)	12. Stereoacuity	13. Perceived demands (training)	14. Perceived demands (testing)	15. Psychological flexibility
1. Training repetitions	28.06(11.12)	–														
2. Total training score	3377.76 (2092.52)	0.967***	–													
3. Total test score	1075.50 (189.04)	0.480**	0.500**	–												
4. NIS score	553.68 (295.98)	0.112	0.142	0.564**	–											
5. Total follow-up score^a^	899.91 (201.28)	0.323	0.343	0.378	0.297	–										
6. 2D vs. 3D viewing mode (training)	0.47 (0.51)	− 0.537**	− 0.528**	0.010	0.169	0.340	–									
7. 2D vs. 3D viewing mode (testing)	0.56 (0.50)	− 0.087	− 0.063	− 0.526**	− 0.699***	− 0.238	0.007	–								
8. Manual dexterity (bimanual)	11.88 (1.70)	− 0.382*	− 0.405*	− 0.349*	− 0.202	− 0.376	0.136	0.150	–							
9. Manual dexterity (assembly)	42.91 (5.57)	− 0.285	− 0.329	− 0.424*	− 0.205	− 0.663**	− 0.114	0.040	0.700***	–						
10. Visual-spatial ability	13.24 (5.07)	− 0.167	− 0.205	− 0.211	− 0.384*	0.024	− 0.080	− 0.089	0.045	0.170	–					
11. Visual acuity (L + R)	5.54 (3.08)	0.084	0.085	0.483**	0.525**	0.080	− 0.128	− 0.367*	− 0.238	− 0.307	− 0.242	–				
12. Stereoacuity	33.38 (18.25)	0.119	0.044	0.017	0.093	− 0.025	− 0.177	0.002	− 0.055	− 0.194	0.099	.378*	–			
13. Perceived demands (training)	53.60 (13.13)	0.410*	0.434*	0.153	− 0.086	0.267	− 0.091	0.005	− 0.278	− 0.065	0.217	− 0.127	− 0.295	–		
14. Perceived demands (testing)	56.95 (13.44)	0.079	0.074	0.187	0.364*	0.375	0.235	− 0.230	− 0.173	0.007	0.115	0.134	− 0.111	0.660***	–	
15. Psychological flexibility	28.97 (7.63)	− 0.432*	− 0.430*	− 0.363*	− 0.144	0.037	0.160	0.154	− 0.098	0.176	0.198	− 0.141	− 0.366*	0.203	0.268	–

*N* = 34

^a^*N* = 23

*p < 0.05, **p < 0.01, ***p < 0.001. *Note*. Bolded items reflect significant relationships at *p* < 0.05.

**Table 3:** Summary of combined hierarchical stepwise regression analyses for variables predicting total training repetitions

Total training repetitions	Model 1 (Control)			Model 2 (Final)			*R*^2^	$R_{{{\text{adj}}}}^{2}$	*R*^2^ change
Variable	*B*	SE	*β*	*B*	SE	*β*			
Constant	33.61	2.25	–	54.77	11.75	–			
2D vs. 3D viewing mode (training)	− 11.80	3.27	− 0.54**	− 8.53	2.38	− 0.39**	0.289	0.267	0.289**
Perceived demands (training)				0.34	0.10	0.40**	0.420	0.383	0.131*
Psychological flexibility				− 0.69	0.16	− 0.48***	0.615	0.577	0.195**
Manual dexterity (bimanual)				− 1.74	0.72	− 0.27*	0.679	0.635	0.064*

*N* = 34

*p < 0.05, **p < 0.01, ***p < 0.001. *Note*. Bolded items reflect significant relationships at *p* < 0.05.

**Table 4:** Summary of combined hierarchical stepwise regression analyses for variables predicting total training score

Total training score	Model 1 (control)			Model 2 (final)			*R*^2^	$R_{{{\text{adj}}}}^{2}$	*R*^2^ change
Variable	*B*	SE	*β*	*B*	SE	*β*			
Constant	4404.78	425.23	–	8529.96	2127.83	–			
2D vs. 3D viewing mode (training)	− 2182.40	619.87	− 0.53**	− 1543.90	430.76	− 0.37**	0.279	0.257	0.279**
Perceived demands (training)				66.75	17.17	0.42**	0.429	0.392	0.150**
Psychological flexibility				− 132.46	28.91	− 0.48***	0.629	0.592	0.200***
Manual dexterity (bimanual)				− 350.59	130.86	− .29*	0.703	0.662	0.074*

*﻿N* = 34

*p < 0.05, **p < 0.01, ***p < 0.001. *Note*. Bolded items reflect significant relationships at *p* < 0.05.

## Results

### Participants

Overall, participants reported a *High* mean interest in surgery (compared to the average medical student) (*M* = 4.00, *SD* = 0.82). Participants’ mean scores across all other variables of interest are shown in Table 2. There were no significant differences in the characteristics, survey responses, or performances during training or testing between participants who completed the follow-up measures online in the lab vs outside the lab (all *p’*s > 0.05). Furthermore, there were no changes in any of the relationships between variables (in significance or direction) after the additional responses/performances were included from these participants.

### Training

#### Repetitions

Bivariate correlations showed significant relationships between total training repetitions and viewing mode (in training), psychological flexibility, perceived demands (in training), and manual dexterity (bimanual) (see Table 2). Equally, the final model to predict total training repetitions included training viewing mode (control variable), perceived demands (training), psychological flexibility, and manual dexterity (bimanual). All associated analyses are shown in Table 3. The final model was statistically significant, *F*(4, 29) = 15.33, *p* < 0.001, and explained 63.5% of the variance in total training repetitions, $R_{{{\text{Adj}}}}^{2} = 0.635$ (large effect). When controlling for the effects of other predictors, the results showed that as psychological flexibility increased, the number of training repetitions required to reach proficiency significantly decreased (i.e. performance improved). Additionally, as perceived demands increased, the number of training repetitions significantly increased (i.e. performance worsened), and as manual dexterity (bimanual) improved, training repetitions significantly decreased. Overall, after controlling for viewing mode, psychological flexibility uniquely accounted for 19.5% of the variance in total training repetitions, while perceived demands accounted for 13.1%, and manual dexterity (bimanual) accounted for 6.4%.

#### Total Training Score

Bivariate correlations showed significant relationships between total training score and viewing mode (in training), perceived demands (in training), psychological flexibility, and manual dexterity (bimanual) (see Table 2). Equally, the final model to predict total training score included training viewing mode (control variable), perceived demands (training), psychological flexibility, and manual dexterity (bimanual). All associated analyses are shown in Table 4. The final model was statistically significant, *F*(4, 29) = 17.12, *p* < 0.001, and explained 66.2% of the variance in total training score*,* $R_{{{\text{Adj}}}}^{2} = 0.662$ (large effect). When controlling for the effects of other predictors, the results showed that as psychological flexibility increased, the total training score (time + errors) significantly decreased (i.e. performance improved). Additionally, as perceived demands increased, the total training score significantly increased (i.e. performance worsened), and as manual dexterity (bimanual) improved, total training score significantly decreased. Overall, psychological flexibility uniquely accounted for 20% of the variance in total training score, perceived demands accounted for 15%, and manual dexterity (bimanual) accounted for 7.4%.

### Testing

Bivariate correlations showed significant relationships between total test score and viewing mode (in testing), visual acuity, manual dexterity (bimanual), manual dexterity (assembly), and psychological flexibility (see Table 2). Despite this, the final model to predict total test score included test viewing mode (control variable) and manual dexterity (assembly) only. All associated analyses are shown in Table 5. The final model was statistically significant, *F*(2, 31) = 12.15, *p* < 0.001, and explained 40.3% of the variance in total test score*,* $R_{{{\text{Adj}}}}^{2} = 0.403$ (medium-to-large effect). When controlling for the effects of test viewing mode, the results showed that as manual dexterity (assembly) increased, the total test score (time + errors) significantly decreased (i.e. performance improved), with manual dexterity (assembly) uniquely accounting for 16.3% of the variance in test performance.

**Table 5:** Summary of combined hierarchical stepwise regression analyses for variables predicting total test score

Total test score	Model 1 (control)			Model 2 (final)			*R*^*2*^	$R_{{{\text{adj}}}}^{2}$	*R*^2^ change
Variable	*B*	SE	*β*	*B*	SE	*β*			
Constant	1185.80	42.15	–	1770.44	198.58	–			
2D vs. 3D viewing mode (testing)	− 197.38	56.38	− 0.53**	191.37	50.47	− 0.51**	0.277	0.254	0.277**
Manual dexterity (assembly)				− 13.70	4.57	− 0.40**	0.439	0.403	0.163**

*N* = 34

*p < 0.05, **p < 0.01, ***p < 0.001. *Note*. Bolded items reflect significant relationships at *p* < 0.05.

### Novel task (NIS)

Bivariate correlations showed significant relationships between total NIS score and viewing mode (in testing), visual acuity, visual-spatial ability, and perceived demands (in testing) (see Table 2). However, the final model to predict total NIS score included test viewing mode (control variable), visual-spatial ability, and perceived demands (testing). All associated analyses are shown in Table 6. The final model was statistically significant, *F*(3, 30) = 30.68, *p* < 0.001, and explained 73.0% of the variance in total NIS score, $R_{{{\text{Adj}}}}^{2} = 0.730$ (large effect). When controlling for the effects of test viewing mode, the results showed that as visual-spatial ability increased, the total NIS score (completion time) significantly decreased (i.e. performance improved). Additionally, as perceived demands increased, the total NIS score increased (i.e. performance worsened). Overall, visual-spatial ability uniquely accounted for 20.1% of the variance in NIS task performance, while perceived demands accounted for 6.4%.

**Table 6:** Summary of combined hierarchical stepwise regression analyses for variables predicting total NIS score

Total NIS score	Model 1 (Control)			Model 2 (Final)			*R*^*2*^	$R_{{{\text{adj}}}}^{2}$	*R*^2^ change
Variable	*B*	SE	*β*	*B*	SE	*β*			
Constant	783.20	55.47	–	816.00	143.55	–			
2D vs. 3D viewing mode (testing)	− 410.73	74.20	− 0.70***	− 400.10	54.74	− 0.68***	0.489	0.473	0.489***
Visual-spatial ability				− 27.72	5.33	− 0.48***	0.690	0.670	0.201***
Perceived demands (testing)				5.76	2.06	0.26**	0.754	0.730	0.064**

*N* = 34

*p < 0.05, **p < 0.01, ***p < 0.001. *Note*. Bolded items reflect significant relationships at *p* < 0.05.

### Follow-up tasks

Bivariate correlations showed a significant relationship between follow-up score and manual dexterity (assembly) (see Table 2). Consequently, the final model to predict total follow-up score included manual dexterity (assembly) alone. Associated analyses are shown in Table 7. The final model was statistically significant, *F*(1, 21) = 16.50, *p* = 0.001, with manual dexterity (assembly) uniquely explaining 41.3% of the variance in total follow-up score*,* $R_{{{\text{Adj}}}}^{2} = 0.413$ (medium-to-large effect). The results showed that as manual dexterity (assembly) improved, the total follow-up score (time + errors) significantly decreased (i.e. performance improved).

**Table 7:** Summary of regression analysis for variables predicting total follow-up score

Total follow-up score	Model 1 (Final)			*R*^*2*^	$R_{{{\text{adj}}}}^{2}$	*R*^2^ change
Variable	*B*	SE	*β*			
Constant	1895.26	247.11	–			
Manual dexterity (assembly)	− 23.15	5.70	− 0.66**	0.440	0.413	0.440**

*N* = 23

*p < 0.05, **p < 0.01, ***p < 0.001

## Discussion

The aim of this study was to expand on past research and assess the impact of physical, cognitive, visual, and psychological variables on novices’ performance in basic laparoscopic skills training and testing. Results indicated that (a) manual dexterity, (b) visual-spatial ability, (c) visual abilities (i.e. visual acuity), (d) perceived demands, and (e) psychological flexibility were all independently correlated with at least one aspect of performance during proficiency-based laparoscopic skills training and/or subsequent testing. However, when collectively assessing the variables and controlling for the well-known effects of viewing mode (i.e. 2D vs 3D) on performance, the results suggest that certain psychological functions (i.e. psychological flexibility and perceived demands) were key to efficiently overcoming the initial challenges involved in training to proficiency. Then, once these challenges were met (i.e. proficiency was achieved and participants were performing at a relatively equivalent level), innate abilities appeared to take centre stage and exert a greater influence on subsequent performance. Despite this, the value of each individual ability did not remain equivalent across each task and stage of practice. For example, we found that basic two-handed dexterity was more predictive of performance during training (when participants were still becoming familiar with the basic movements and tasks), while the two-handed assembly task (requiring more complex, simultaneous, and continuous movements with multiple elements) was more predictive of performance in later testing of pre-practiced and new (more complex) FLS tasks. It must be noted that, to the authors’ knowledge, the various aspects of *manual* *dexterity* have never been broken down in previous studies to explore how they respectively impact performance during the different stages of laparoscopic skills training. This may help to explain why inconsistent outcomes have been identified in past research, as manual dexterity has been conceptualised and measured in different ways (e.g. performance on a laparoscopic task, or a total score on a grooved pegboard test [16, 17]).

Furthermore, while visual-spatial ability did not account for a significant portion of variance in participants’ training performance (beyond that of physical ability and psychological perception), it explained a significant portion of variance in performance when the task increased in spatial complexity (i.e. in the NIS task). Similarly, while perceived task demands (in testing) did not account for any significant difference in the final model predicting participants’ test performance (of pre-practiced tasks), these perceptions did predict a significant portion of variance in performance when the task was novel and highly complex (i.e. the NIS task). Overall, these findings reiterate the limitations inherent in trying to predict surgical skill through one or two innate abilities alone. Rather, performance should be understood as the combination of physical, cognitive, visual, and psychological skills/abilities that work together to allow effective movement, attention, observation, and emotional regulation that changes with the needs of the context/task/individual. As past research has commonly focussed on only one or two innate variables to predict surgical performance (and overlooked the combined effects of psychological factors) [20, 21], it is not surprising then that we find a lack of consistency in the strength and significance of various relationships throughout the field.

### Limitations and future research

Despite the novelty of the current findings, the study had its limitations. First, though the sample was appropriately powered for our interpretation of the reported analyses, future studies may benefit from employing larger sample sizes to allow for greater generalisability of the results and to reduce the possibility of Type II errors. While no substantive conclusions were drawn from non-significant relationships here, several findings appear inconsistent with the outcomes of prior research. For example, unlike in previous studies [63], stereoacuity was not significantly correlated with performance, and visual acuity did not account for a significant portion of variance in performance beyond other innate variables. However, these results may have been due to a restricted range of responses (i.e. all participants were within the normal range of visual ability), and a level of shared variance with other predictors (e.g. viewing mode, manual dexterity). Consequently, future research should explore the effects of visual abilities across a broader range and a larger sample of participants.

Additionally, while the results highlight the need to expand on previous models to optimise the prediction of surgical potential, several variables were omitted from the study’s design that may have accounted for further variance in participant’ performance. For example, the NASA-TLX was used to measure how demanding participants perceived the tasks in this study, yet no specific measure of stress perception (i.e. perceiving the task as a *threat* or a *challenge*) was included here. Therefore, we were not able to determine whether psychological flexibility impacted performance via different stress perceptions. Furthermore, assessing other subcomponents of psychological flexibility, namely, *cognitive* *flexibility* (an aspect of executive functioning that allows flexible response adaptation and attention shifting, which has been found to correlate with psychological flexibility in other contexts) [75] may have accounted for further variance in the outcome measures and helped to explain the connections found between psychological variables and participant performance here.

In addition, as this study focussed on initial training and the early acquisition of skills, we cannot determine whether the value of psychological flexibility is limited to overcoming the initial challenges and frustrations involved in early training, or whether there are broader implications for practice. For example, if such results are found in a controlled training environment, it begs the question: What kind of impact could psychological flexibility have on the performance of early career surgeons who continuously face challenges, uncertainty, and extreme demands while practicing in the high-stakes operational context? Consequently, further exploration is required into how the potential for extreme stress (i.e. high perceived task demands, threat perceptions, and/or low psychological flexibility) in early training impacts performance in the later stages of training and performance in the real operational context. Thankfully, unlike innate abilities that can be particularly difficult to change/improve over time, research shows that psychological flexibility is a skill that may be taught and/or increased through mindfulness-based practice [76–79]. Furthermore, as mindfulness-based practice has already been linked to improved well-being and performance in laparoscopic skills training [60], the combined assessment of psychological flexibility and mindfulness may help to broaden our understanding of performance and establish more effective and individualised tools to prepare potential candidates for a surgical career.

## Conclusion

Overall, the current results highlight the importance of assessing physical, cognitive, visual, and psychological functioning to predict surgical potential. Moreover, this research provides a novel insight into the value of psychological functioning to increase the efficiency of proficiency-based laparoscopic skills training. With further exploration into these and other variables of interest (e.g. cognitive flexibility, attention, mindfulness, stress perceptions), we may be able to develop more reliable tools/guides to enhance the self-selection and self-development of surgical candidates. In doing so, these candidates may be more likely to possess the necessary aptitude and attitude to reach laparoscopic proficiency with increased efficiency and efficacy, with benefits for surgeons, patients, and the healthcare system alike.
